# Nutritional profiling and contaminant levels of five underutilized fish species in Norway

**DOI:** 10.3389/fnut.2023.1118094

**Published:** 2023-03-08

**Authors:** Sophie Kendler, Frida Walle Thornes, Anita Nordeng Jakobsen, Jørgen Lerfall

**Affiliations:** Department of Biotechnology and Food Science, Norwegian University of Science and Technology (NTNU), Trondheim, Norway

**Keywords:** nutritional profile, flatfish, underutilized fish, DIAAS, omega 3 fatty acids, risks and benefits, healthy

## Abstract

Exploring and making use of underutilized marine resources can be a sustainable approach to achieve future demands of fish consumption by the ever-growing population. Five species, namely European plaice (*Pleuronectes platessa*), European flounder (*Platichthys flesus*), lemon sole (*Microstomus kitt*), megrim (*Lepidorhombus whiffiagonis*), and thornback ray (*Raja clavate*), often captured as by-catch in Norway, were characterized for their nutritional value and potential accumulation of hazardous components. The proximate composition, protein profile, fatty acid profile as well as essential and toxic trace elements and polychlorinated biphenyls (PCBs) were analyzed. Digestible indispensable amino acid (DIAA) ratios and scores (DIAAS) and contributions of omega-3 fatty acids to the diet were calculated. Analysis on proximate composition revealed low fat contents of 0.74 to 1.25% and sufficient protein contents between 16.9 and 24% in the five species. Results of DIAA indicate a profitable distribution, with contributions exceeding the daily intake recommendations for an adult person related to a 200 g fillet. Moreover, findings on the distribution of eicosapentaenoic (EPA) and docosahexaenoic acid (DHA) showed remarkable results, considering that the investigated species are lean fish. All five investigated fish exceed the recommended average daily intake level (AI) of EPA + DHA in a 200 g portion. As to toxic trace elements and PCBs, no significantly elevated levels were found considering a portion size of 200 g. Consequently, the nutritional quality of the investigated fish can be regarded as profitable with overall low potential health risks.

## 1. Introduction

By 2050, the demand for food is projected to increase by 50% as the world’s population is expected to reach 9.7 billion people (1, 2). At the same time, food production accounts for a quarter of total greenhouse gas emissions, and immediate action is needed to reduce climate gas emissions and counter climate change (3). Finding ways to produce more food, while at the same time reducing the climate impact of food, is a tremendous challenge in the years to come. Norway, being the country with the second-longest coastline globally shows a variety of marine species. Moreover, Norway is the second largest fish exporter (4). Nevertheless, only around 10% of Norway’s 220 marine species have been commercially utilized as food (5). A sustainable approach to achieve the future demand for fish can be to explore species that are considered as underutilized or classified as little utilized resources (LUR). A report on LUR species in Norway was published in 2011 and concluded that flatfish were among the species with the most significant potential for successful commercialization (6). Further research and investigations on flatfish were recommended to enable commercialization (6).

The excellent nutritional composition of fish and seafood in general has been reported in various studies and reviewed amongst others by Khalili Tilami et al. (7). Moreover, authorities have set recommendations to guarantee a satisfying intake of certain nutrients. FAO/WHO (8) and the European Food Safety Authority (EFSA) recommend a seafood consumption of 100 g and up to 300 g of fish per week, respectively, accounting for at least two meals a week to cover the recommended intake. High amounts of important long chain polyunsaturated fatty acids (LC-PUFAs) in fish are recognized for promoting overall human health due to their activities in physiological, molecular as well as cellular processes (9). Furthermore, marine proteins are recognized for their favorable nutritional value, due to high bioavailability and abundance of important peptides and essential amino acids. Studies focusing on the potential health benefits of marine proteins and hydrolysates are becoming increasingly prevalent due to their beneficial digestibility (7). Khalili Tilami et al. (7) mention that results indicate that fish proteins, peptides, and hydrolysates give improved health benefits somewhat comparable to marine lipids.

Next, to being a source of valuable macronutrients, fish contain essential trace elements like calcium and selenium. As a key indicator of bone density, calcium is crucial for the health of the skeleton and plays a vital role in many metabolic processes. Moreover, selenium deficiencies can lead to several diseases (7). Next to the importance of maintaining metabolic health in humans, selenium in fish is especially important because of its potential counter effects on methylmercury (10). Methylmercury is the methylated form of mercury, which naturally occurs in, e.g., volcanos and the atmosphere, but can also end up in environmental cycles if human caused sources like, e.g., fungicides, antiseptics or batteries are inappropriately discarded (11). Methylmercury is known to have several harmful impacts on human health. It is particularly problematic for pregnant women, as it can migrate across the placenta walls. High methylmercury exposure in pregnant women directly affects the neurodevelopment of the fetus (12). Moreover, bioaccumulation of persistent organic pollutants such as, e.g., polychlorinated biphenyls (PCBs) can pose serious health issues due to their persistence and toxicity to the human body (13).

The aim of the present study was to characterize four different flatfish (*Pleuronoectiformes*) species, and a ray (*Rajiformes*), whereof all are often captured as by-catch in Norway and regarded as underutilized species. More specifically, the study presents the chemical and nutritional profile of European plaice (*Pleuronectes* platessa), European flounder (*Platichthys flesus*), lemon sole (*Microstomus kitt*), megrim (*Lepidorhombus whiffiagonis*), and thornback ray (*Raja clavate*), including comparisons between species related to health promoting nutritional components. The analyses contained the proximate composition, total and free amino acids as well as fatty acid profile. Marine food sources in general are regarded as the main contributors to the intake of contaminants, which pose a potential risk for the consumers. Therefore, PCBs and trace elements, including both essential and toxic elements, were determined to support the safe consumption of these species.

## 2. Materials and methods

### 2.1. Raw material

**Figure 1:**
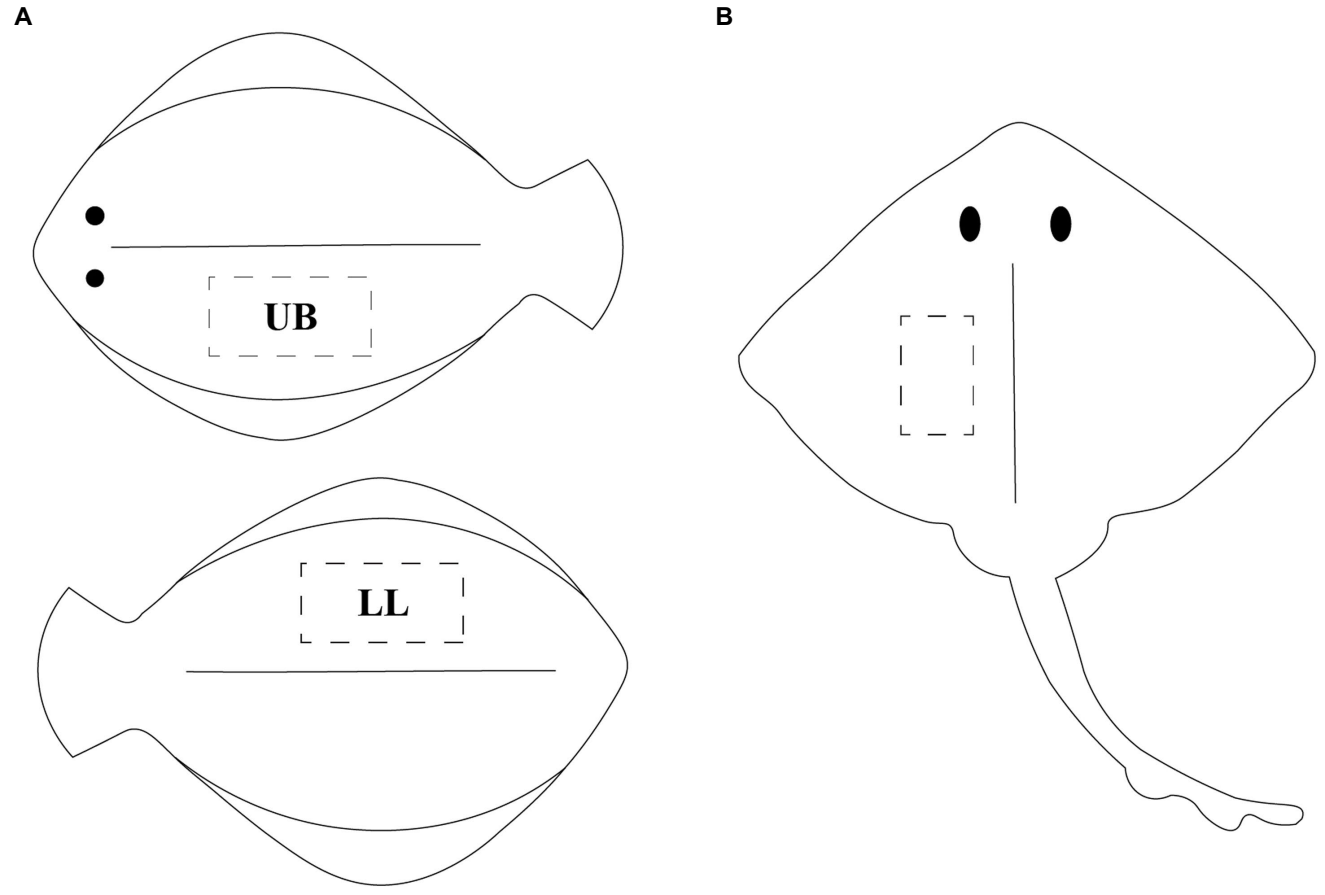
**(A)** Graphical illustration of flatfish, visualizing the two sampling points upper belly (UB) on the upper body part and lower loin (LL) on the back side of the fish; **(B)** illustration of ray, visualizing the sampling point of the central fillet.

The five fish species of interest were captured with purse seine between September 2020 and April 2021 by local fishermen at the Norwegian west coast. The catch occurred in area 2.a.2, according to the FAO Major Fishing Areas (14). The fish was gutted immediately after capture and kept on ice until the end of rigor mortis (41–57 h post-mortem). Fish were either filleted directly or frozen as whole (−80°C) and subsequently thawed before filleting. In order to have a thorough understanding of the chemical composition and identify any potential changes in the nutritional content within different body regions, muscle samples were taken from two fillets per flatfish. The two fillets, being the lower loin (LL) and upper belly (UB), of the flatfish samples were considered for analyses (*n*_Flounder_ = 7 × 2, *n*_Lemon sole_ = 5 × 2, *n*_Plaice_ = 10 × 2, *n*_Megrim_ = 5 × 2), as shown in Figure 1A. In contrast, only one fillet was kept for thornback ray (*n* = 5), as it consists of two central fillets (Figure 1B). All fillets were frozen directly and stored at –80°C until further use.

### 2.2. Proximate composition

Dry matter and ash content was determined following the AOAC 925.10 method (15). Samples were homogenized and between 1 and 2 g were weighed in duplicates in porcelain crucibles. The samples were placed in a dehydrator at 105°C for 24 h (TS8056; Termaks, Norway). After 24 h, the samples were placed in a desiccator to cool down to room temperature, weighed and water content was calculated according to equation 1. The dried samples were transferred to an ash oven and burned at 550°C for 20 h (B410; Nabertherm,Germany). The samples were placed in a desiccator to cool down, weighed and the inorganic matter was then determined following the principle of equation 1.

(1) $$Watercontent\;\left(\%\right)=\frac{Sample_{wet}-Sample_{dried}}{Sample_{wet}}\times 100$$

Total crude protein content (%) was determined using the Kjeldahl method (15). A Kjeldahl apparatus (K-449 and K-375, Büchi Labortechnik, Schwitzerland) was used for measurements. The sample digestion and titration were carried out following the application manual No: 114/2013 of Büchi Switzerland. Briefly, sulfuric acid (H_2_SO_4_, 95–97%) and two Kjeldahl Tablets Eco (3.5 g K_2_SO_4_/0.105 g CuSO_4_ × 5H_2_O/0.105 g TiO_2_) were added to samples (1.5 g) before digestion. Digested samples were first neutralized with NaOH (32%, 15–90 ml) and H_2_SO_4_ (0.25 mol/l) was used as the titration solution subsequently. To determine the total protein concentration, a conversion factor of 6.25 × nitrogen (%) was applied (16).

Total lipids (%) were determined following the method of Bligh et al. (17). Samples (2 g ww) were weighed into chloroform-resistant tubes and chloroform was added. The solvent-sample mixture was subjected to extensive homogenization and centrifugation to achieve phase separation. The aqueous phase and chloroform-lipid phase were separated. Chloroform was evaporated from samples by applying liquid nitrogen. To accelerate the evaporation, the samples were placed on a heating block turned to 40°C (Stuart™ block heater type: SBH130D/3, Cole-Parmer, United States). The total lipids (%) were calculated according to Bligh et al. (17). The remaining chloroform phase containing lipids was frozen and stored at −80°C for fatty acid analysis.

### 2.3. Fatty acid composition

Fatty acids were prepared as methyl esters for analysis by gas chromatography. For fatty acid methyl ester (FAME) preparation, the method of Metcalfe et al. (18) was used. Chloroform phases containing lipids from individual fish were systematically merged to obtain five samples for thornback ray (*n* = 5), four samples for megrim (*n* = 4), and three samples each for lemon sole (*n* = 3) and flounder (*n* = 3). As these fish are very lean and individual fish were limited in size, it was necessary to merge samples to obtain at least 0.02 g of lipids per sample. Samples from European plaice were not merged due to bigger fish sizes, and 6 individuals (*n* = 6) were chosen for analysis. Nitrogen evaporation was conducted at 30°C until all chloroform was removed from the samples, and 3 ml KOH in methanol (0.5 M) was added to the samples and vortexed to saponify the lipids. Samples were incubated in a water bath at 70°C for 20 min, vortexed, and cooled on ice. Afterwards, 5 ml of boron trifluoride-methanol (14%, BF_3_) was added to allow acid-catalyzed esterification of the fatty acids. The samples were re-incubated in the water bath at 70°C for 5 min and cooled on ice. *N*-butyl acetate (2 ml) was added, and the samples were shaken. Subsequently, saturated NaCl (around 1.5 ml), and two spatulas of powdered sodium sulfate (Na_2_SO_4_) were added to the samples, and the samples were rested at room temperature (21°C) to allow phase separation. Around 0.5 ml of hexane was added, and the lipid phase was then pipetted out and filtered using a 0.2 μm PTFE membrane (VWR International, United States) into GC vials.

The FAMEs were analyzed by gas chromatography (GC) using a GC apparatus (Agilent 6850, Agilent Technologies, United States). The samples (2–3 μl) were introduced by an evaporation injector (inlet: 260°C, pressure: 18.1 psi). Hydrogen was used as a carrier gas to pass the samples onto a polyethylene glycol column (HP-INNOWAX, i.D.: 0.25 mm; film: 0.25 μm, Merck Life Sciences, Norway), where FAMEs were separated at different times along the stationary phase. A flame ionization detector (FID) adjusted to 310°C was used to detect the samples. The oven program was set to a constant temperature of 160°C for 3 min, with an increase of 3°C/min to 240°C and held for 3 min.

Fatty acids were identified by comparing relative retention times (RRTs) of the external FAME standard mix containing 37 fatty acid methyl esters (Supelco 37 Component FAME Mix, Merck Life Sciences, Norway) with sample peaks. Chromatogram peaks, showing similar RRTs to the external standard were considered for determination. The intensity of each peak was calculated against the total intensity of FAMEs, to determine the percentage distribution of the individual fatty acids in each sample.

### 2.4. Protein profile

#### 2.4.1. Amino acid distribution

Total amino acids were extracted from the samples following the method of Blackburn (19). Samples were freeze-dried for 22 h at −40°C and 13.3 Pa as a preparation for the analysis. Freeze-dried samples (80 mg) were weighed up in duplicates, and 1 ml of HCl (6 M) was added. The tubes were incubated for 22 h at 105°C to allow protein hydrolysis. Hydrolyzed samples were pH-neutralized by adding NaOH. The samples were filtered through a glass microfiber filter GF/C using suction, subsequently filled up to 10 ml with deionized water and suitably diluted. Diluted samples were filtered through 0.22 μm polyethersulfone filters (VWR International, United States) and transferred into HPLC vials.

Both free and total amino acids were analyzed by ultra-high-performance liquid chromatography (HPLC, UltiMate 300, Thermo Fisher Scientific, United States). As mobile phase, methanol and sodium acetate (0.08 M) with 2% tetrahydrofuran were applied. The HPLC was equipped with a Nova-Pak C18 column (WAT086344, particle size: 4 μm, 3.9 mm*150 mm, Waters Corp., United States), a TSP P400 pump and an injection valve (ultimate 3000WP injector). A pre-column derivatization step using the *o*-phtalaldehyde (OPA) method was applied and the flow rate was adjusted to 0.9 ml/min. After passing through the column, the amino acids were detected by fluorescence and recognized by a Dionex RF2000 detector. Alpha-aminobutyric acid (Aba) was used as an internal standard. Three amino acids were not analyzed: cysteine, proline, and tryptophan. The amino acids glycine and arginine were co-eluted in the analysis.

#### 2.4.2. Free amino acid distribution

Free amino acids were extracted from the samples following the method of Osnes et al. (20). Approximately 2 g of frozen grated sample was placed into centrifuge tubes. Deionized water (10 ml) was added to the tubes, and the mixture was homogenized for 45 s to disrupt cells and release proteins (Ultra Turrax T25, Ika, Germany). The tubes were centrifuged for 3 min at 500 g at 4°C to obtain two phases (1700, Kubota, Japan). The soluble protein extract phase was taken out, and 1 ml of the extract was mixed with 0.25 ml of sulphosalicylic acid (10%, C_7_H_6_O_6_S) to allow protein breakdown. The samples were shaken vigorously and placed in a fridge (4°C) for 30 min. After protein breakdown, the tubes were centrifuged for 10 min at 2700 g and 4°C (Megafuge 8R, Thermo Fisher Scientific, United States). The supernatant containing the free amino acids was suitably diluted. The diluted samples were filtered through 0.2 μm polyethersulfone membrane filters and 0.205 ml of the samples were transferred to vials before performing HPLC analysis as described in section 2.4.1.

### 2.5. Trace elements and polychlorinated biphenyls

For analyzing potentially elevated levels of contaminants in the samples, a variety of trace elements and polychlorinated biphenyls (PCBs) were chosen, and samples were pooled together. Each sample contained two individuals (three for European plaice) of same size, equally distributed and homogenized. Per species, two pooled samples (*n* = 2) were examined.

The samples were analyzed for 20 elements, including both toxic and essential trace elements such as Ag, Al, As, Ca, Cd, Co, Cr, Cu, Fe, Hg, K, Mg, Mn, Mo, Na, Ni, Pb, Se, V, and Zn. An inductive coupled plasma mass spectroscopy (ICP-MS, 8800 Triple Quadrupole; Agilent Technologies, United States) system was used. The system was linked to an autosampler (prepFAST M5, ESI, United States). To test the accuracy of the analysis, certified reference materials (CRM) were used, namely MODAS-5 (cod tissue, Nr. 0496) and MODAS-3 (herring tissue, Nr. 0958). The procedure of sample preparation, including microwave digestion and subsequent steps were previously described in detail by Kendler et al. (21) following the method of Sørmo et al. (22).

The analysis for PCBs included PCB-3, 8, 28, 52, 101, 118, 138, 153, 180, 195, 206, and 209, including the ICES-6 PCBs (PCB: 28, 52, 101, 138, 153, 180) and the dioxin-like PCB 118. A GC–MS system (7890A, Agilent Technologies, United States) was employed to detect PCBs. The system included split liner injection, an inert mass selective detector (5,975, Agilent Technologies, United States) and a Thermo TG 5MS column (length: 30 m; i.D.: 250 μm; film: 0.5 μm). A detailed description of the procedure can be found in Kendler et al. (21). The sample extraction followed the method described by Teunen et al. (23).

### 2.6. Nutritional quality parameters

#### 2.6.1. Digestible indispensable amino acid score

The protein quality of a foodstuff can be determined by calculating the digestible indispensable amino acid score (DIAAS). For calculations on the quality of the distinct amino acid profiles of the five investigated fish species, the DIAAS as proposed by FAO (24) was considered and calculated.

The score is a product of the amino acid scoring pattern of the protein and the digestibility of these amino acids. The amino acid scoring pattern is related to how the amino acids in the protein correspond to the nutritional requirements set by FAO (24). A beneficial amino acid content is characterized by a high digestible indispensable amino acid (DIAA) content, which exceeds the nutritional requirements.

The DIAA reference ratios can be calculated for each DIAA from the amino acid content and the ileal digestibility, as seen in equation 2. IAA ratios above one are characterized by a high content of DIAA, which exceeds nutritional recommendations. DIAA ratios below one mean that the DIAA in the protein does not meet the recommendations. The lowest DIAA reference ratio is multiplied by 100 to obtain the DIAAS (24). Food with scores above 100 can be classified as “excellent” protein quality sources, scores between 75 and 100 can be classified as “good” protein quality sources, while scores below 75 can be regarded as “low” protein quality sources (24). Previous investigations on DIAAS in fish have determined them to be of excellent protein quality (25, 26).

(2) $$DIAAreference\;ratio=\frac{mg\;of\;AA\;in\;1\;g\;sampleprotein\times df}{mg\;of\;AA\;in\;1\;g\;ofreferenceprotein}$$

Where:

df: true ileal digestibility factor for specific amino acids in fish as proposed by FAO (27). When specific digestibility factors were not available for the given amino acid, the general digestibility factor for the protein was used.

reference protein: nutritional requirements set by FAO et al. (28).

#### 2.6.2. Fatty acids

An estimation of the total amounts of fatty acids per 100 g edible fillet wet weight (ww) of the investigated species was conducted using equation 3. For the assessment of total fatty acids, published work from Weihrauch et al. (29) considering different lipid conversion factors in fish was applied, following the fatty acid conversion factor (FACF) as shown in equation 4.

(3) $$\begin{array}{l} g\;fattyacid\;per\;100\;g\;fillets=weight\%\;\;FAME\times FACF\\ \times \;\;TLC \end{array}$$

Where:

Weight% FAME: results from FAME analysis, assuming the same as weight%-FA since marine lipids mainly consist of long-chain fatty acids (29).

FACF: fatty acid conversion factor (g FA/g lipid), from conversion factors proposed by Weihrauch et al. (29) calculated as in equation 4.

(4) $$FACF=\frac{0.933-0.143}{TLC}$$

TLC: total lipid content as measured in g lipid per g fillet ww from the analysis on total lipids (17).

### 2.7. Statistical analysis

All statistical analyses were performed using Minitab 19^1^ (Minitab Inc., United States). A Grubbs Outlier test with a significance level of *α* < 0.05 was conducted to find outliers in the data set. Data were analyzed using univariate analysis of variance (ANOVA) combined with Tukey HSD *post hoc* test when significance was detected to investigate the differences between groups. Statistical differences were reported at the level of *α* < 0.05. For flatfish representatives, analyses were carried out in 2 × 2 parallels (2 parallels for each UB and LL fillet; 4 in total per sample) and are presented as means ± standard deviation (SD) if not other stated. For thornback ray, the same analyses were performed in duplicates.

## 3. Results and discussion

In addition to species comparison, differences in the proximate and total and free amino acids composition among the UB and LL fillets were investigated for the four flatfish species. The UB fillets did not significantly differ from the LL for any of the species (*p* > 0.05). Hence, the data of the UB and LL fillets were combined, giving one mean value for each flatfish individual, which was further considered when presenting the results. This leads to the assumption that nutrients are equally distributed throughout the body regions of the investigated flatfish species. The findings correspond with our previous study on European plaice (21), where no significant difference in proximate and nutritional composition between muscle samples from upper and lower body fillets was found. Moreover, the results are in accordance with the study of Barbosa et al. (30) on megrim, which found no differences in lipid content between the upper and lower body fillets. The differences between fillets of upper and lower body were not investigated for thornback ray as its morphology differs from flatfish species, having only two main fillets.

### 3.1. Proximate composition

The proximate composition of flounder, lemon sole, megrim, plaice and thornback ray are shown in Table 1. Significant differences were observed for the species’ ash, water, protein and lipid content. Megrim was found to have the lowest average water content of 79.2%, being significantly lower than flounder (*p* = 0.003). The measured water content for megrim equaled the values found by Afonso et al. (31), and Barbosa et al. (30), who showed values from 75–79%. Thornback ray showed a similar water content (80.1%) compared to lemon sole (81.4%) and plaice (80.5%), but differed from previous investigations by Colakoglu et al. (32) and Turan et al. (33) with water contents of 77%. The water contents of all investigated species are similar to those found by Karl et al. (34), investigating different flatfish species with average values ranging from 78.1 to 82.1%.

**Table 1:** Proximate composition of central fillets of flounder, lemon sole, megrim, plaice, and thornback ray.

	Species					
Composition (%)	Flounder	Lemon sole	Megrim	Plaice	Thornback ray	*p*-value^*^
	*n* = 7	*n* = 5	*n* = 5	*n* = 10	*n* = 5	
Ash	1.11 ± 0.07^b^	1.04 ± 0.05^bc^	1.10 ± 0.04^b^	1.25 ± 0.07^a^	0.94 ± 0.10^c^	<0.001
Water	82.1 ± 1.2^a^	81.4 ± 1.0^ab^	79.2 ± 1.4^b^	80.5 ± 1.4^ab^	80.1 ± 0.8^ab^	0.004
Proteins	16.9 ± 1.0^c^	17.5 ± 0.7^c^	19.6 ± 1.2^b^	17.6 ± 1.1^c^	24.0 ± 1.4^a^	<0.001
Lipids	0.94 ± 0.08^ab^	0.74 ± 0.16^b^	0.98 ± 0.16^ab^	1.25 ± 0.47^a^	0.76 ± 0.08^b^	0.015

Results presented as mean values ± SD. *ANOVA was applied to detect differences in proximate composition; where significant difference was detected (*α* < 0.05), a Tukey HSD *post hoc* test was applied. Values with different superscript (a, b) within a row are significantly different (*P* < 0.05).

The ash content of the four flatfish representatives was between 1.0–1.25%, while fillets from thornback ray (0.9%) showed lower values of inorganic material. This was in line with previous investigations on flatfish, although previous studies on thornback ray found slightly higher ash values (1.1–1.4%) for this species (32, 33). Ash content in plaice is higher than previously investigated by Karl et al. (34) of 0.9% but similar to results from three different seasons by Kendler et al. (21) of values ranging from 1.07 to 1.28%. Plaice has a significantly higher ash content compared to flounder (*p* = 0.005) and thornback ray (*p* < 0.001).

For protein content, the differences were more significant between the investigated species. Megrim had a significantly higher protein content than lemon sole (*p* = 0.036), flounder (*p* = 0.003) and plaice (*p* = 0.024) with an average of 19.6%, being marginally higher than the studies by Barbosa et al. (30) with 16.6 to 18.6%. Lemon sole and flounder had a protein content of 16–18%, in line with previous investigations of plaice (16.6%) and yellowfin sole (16.0%) of Karl et al. (34). Significantly higher protein values were observed for thornback ray with 24.0%, being considerably higher than all investigated flatfish species (*p* < 0.001). The measured protein content of thornback ray was not in correspondence with previous studies and was 4–5% higher than observed in studies by Colakoglu et al. (32) and Turan et al. (33) with 18.6 and 20%, respectively. It must be mentioned that the proximate composition of thornback ray exceeds a total of 100%, which indicates an overestimation of the protein content measured by the Kjeldahl method using a conversion factor of 6.25. The Kjeldahl method measures the total nitrogen content, assuming approximately 16% nitrogen in proteins. However, other non-proteins in the cells also contain nitrogen, which can lead to an overestimation of the protein nitrogen in the food (35). Ray tissue contains around 350–400 mM urea, a nitrogen containing non-proteinaceous component, which might have been an interfering substance in the Kjeldahl analysis (36). Moreover, as the relative nitrogen content of amino acids fluctuates and the amino acid composition depends on the protein source, the assumption of 16% nitrogen content is rather general. Studies have shown that using a conversion factor of 6.25 can overestimate the total protein content of some foods (35, 37, 38). For this reason, attention has been given to creating conversion factors that are species/ food-specific (37, 38). Nevertheless, the established conversion factor of 6.25 is still widely used being officially recognized by the AOAC as a standard analytical method for protein determination, which makes results better comparable (39). The results suggest that protein contents of the four flatfish species are not overestimated, as the proximate composition (100%) is not notably exceeded and standard deviations are acceptable (1%).

Regarding the lipid content, all species can be considered as lean species, having values below 2%. Plaice was found to have significantly higher lipid values of in average 1.25% compared to lemon sole (*p* = 0.025) with 0.74% and thornback ray (*p* = 0.036) with 0.76% total fat content. The findings in this study show lower lipid contents for megrim (0.98%) compared to the study of Pastoriza et al. (40) finding a lipid content of up to 1.9%. Karl et al. (34) found lower lipid values for plaice of around 0.8%, but similar values of around 1% of other investigated flatfish. In a previous study of Kendler et al. (21), significant differences in the lipid content of plaice depending on fishing season were discovered ranging from 0.75 to 1.55%. For thornback ray, the measured lipid content of 0.76% was marginally higher than the finding of Turan et al. (33) of 0.5%, while much lower than the finding of Colakoglu et al. (32) of 3.4%. Two previous studies on deep-sea fish found that general deep-sea elasmobranchs like thornback ray had a lipid content of around 0.7 to 1.0%, which support the findings of the present study (41, 42).

### 3.2. Protein profile

Total amino acids (TAA) were investigated to determine the nutritional value of the proteins in the fish. The TAA results for the species are given in Table 2. The most abundant TAA for all species were leucine and lysine, as well as glutamic and aspartic acid, under physiological conditions in the form of glutamate and aspartate. The same abundant amino acids were found in an investigation of three flatfish species by Kim et al. (43), although showing higher amounts of glycine than in the present study. The contents of glutamate and aspartate can be regarded as overestimated as glutamine and asparagine were converted to these two amino acids during acid analysis (44). Consequently, asparagine and glutamine were detected in the lowest amounts in all investigated samples. Significant differences (*p* < 0.05) were found for most of the amino acids between the five species. All species have a preferable distribution of indispensable amino acids (IAA), accounting for more than 50% of the TAA distribution. Megrim was found to have significantly higher amounts of IAA (7.24 g/ 100 g; *p* = 0.002) compared to flounder (6.14 g/100 g), lemon sole (6.07 g/100 g), plaice (5.62 g/100 g), and thornback ray (5.88 g/100 g). Furthermore, megrim has comparably more total amino acids (13.80 g/100 g; *p* = 0.002) than the other species. This was also found in three of the four species for the total protein content, with the exception of thornback ray, where an overestimated protein content is suspected. The amino acid determination by Blackburn (19) directly calculates the protein amount by considering only amino acid residues in the analysis and does not take into account possible interfering non-proteinaceous components. Amino acid hydrolysis can therefore be regarded as a good approach to determine the total amino acid content and gives an indication of the protein content of foods. However, the concentration of some of the amino acids might be lowered significantly due to the hydrolysis step prior to HPLC-analysis, which leads to an underestimation of the total protein content (35). Furthermore, it has to be mentioned that due to their instability during acid hydrolysis, cysteine and tryptophan were not determined in the applied method. Consequently, these two amino acids should be analyzed separately if the TAA measurement is used to indicate the total protein content.

**Table 2:** Total amino acid contents (total-AA) in g/100 g central fillets of flounder, lemon sole, megrim, plaice and thornback ray, showing indispensable (IAA) and non-indispensable amino acids (non-IAA).

	Species					
Amino acids	Flounder	Lemon sole	Megrim	Plaice	Thornback ray	
	*n* = 7	*n* = 5	*n* = 5	*n* = 10	*n* = 5	
Indispensable^*^	**Total-AA**					
	g 100 g^−1^	g 100 g^−1^	g 100 g^−1^	g 100 g^−1^	g 100 g^−1^	*p*-value***
Histidine	0.27 ± 0.04^b^	0.31 ± 0.04^ab^	0.36 ± 0.08^a^	0.28 ± 0.06^ab^	0.26 ± 0.007^b^	0.026
Isoleucine	0.57 ± 0.06^b^	0.59 ± 0.01^b^	0.71 ± 0.05^a^	0.53 ± 0.06^b^	0.59 ± 0.01^b^	<0.001
Leucine	1.13 ± 0.12^b^	1.06 ± 0.09^b^	1.32 ± 0.09^a^	1.03 ± 0.11^b^	1.09 ± 0.02^b^	<0.001
Lysine	1.40 ± 0.08^b^	1.37 ± 0.14^b^	1.63 ± 0.09^a^	1.24 ± 0.17^b^	1.26 ± 0.03^b^	<0.001
Methionine	0.46 ± 0.05^a^	0.20 ± 0.04^b^	0.42 ± 0.12^a^	0.37 ± 0.05^a^	0.41 ± 0.04^a^	<0.001
Phenylalanine	0.56 ± 0.06^b^	0.54 ± 0.05^b^	0.68 ± 0.05^a^	0.51 ± 0.05^b^	0.57 ± 0.01^b^	<0.001
Threonine	0.69 ± 0.04^b^	0.70 ± 0.1^b^	0.85 ± 0.04^a^	0.60 ± 0.06^c^	0.69 ± 0.03^bc^	<0.001
Valine	0.63 ± 0.07^ab^	0.58 ± 0.07^b^	0.72 ± 0.09^a^	0.58 ± 0.06^b^	0.58 ± 0.01^b^	0.008
Ʃ IAA	6.14 ± 0.69^b^	6.07 ± 0.78^b^	7.24 ± 0.42^a^	5.62 ± 0.65^b^	5.88 ± 0.11^b^	0.002
**Non-indispensable**						
Asparagine	<0.01 ± <0.01	<0.01 ± <0.01	<0.01 ± <0.01	<0.01 ± <0.01	<0.01 ± <0.01	0.833
Glutamine	0.03 ± 0.007^b^	0.01 ± 0.004^c^	0.05 ± 0.008^a^	<0.01 ± <0.01^c^	0.04 ± 0.01^b^	<0.001
Arg/Gly^**^	0.82 ± 0.04^ab^	0.83 ± 0.1^ab^	0.90 ± 0.08^a^	0.73 ± 0.07^b^	0.77 ± 0.02^ab^	0.004
Tyrosine	0.52 ± 0.06^ab^	0.49 ± 0.04^ab^	0.56 ± 0.1^a^	0.45 ± 0.05^b^	0.41 ± 0.04^b^	0.005
Alanine	0.89 ± 0.1^ab^	0.74 ± 0.16^bc^	1.0 ± 0.15^a^	0.69 ± 0.06^c^	0.79 ± 0.02^bc^	<0.001
Aspartate	1.39 ± 0.15^ab^	1.35 ± 0.15^ab^	1.61 ± 0.13^a^	1.35 ± 0.17^b^	1.32 ± 0.03^b^	0.022
Glutamate	1.79 ± 0.19	1.83 ± 0.20	2.10 ± 0.15	1.95 ± 0.24	1.75 ± 0.05	0.049
Serine	0.74 ± 0.12^ab^	0.85 ± 0.12^a^	0.84 ± 0.14^a^	0.60 ± 0.07^b^	0.62 ± 0.01^b^	<0.001
Ʃ Non-IAA	5.64 ± 0.62^ab^	5.61 ± 0.52^ab^	6.56 ± 0.52^a^	5.34 ± 0.57^b^	5.29 ± 0.11^b^	0.005
Ʃ Total-AA	11.78 ± 1.3^b^	11.68 ± 1.3^b^	13.80 ± 0.9^a^	10.96 ± 1.2^b^	11.17 ± 0.2^b^	0.002

Results presented as mean values ± SD. *Tryptophan is not detected due to acid hydrolysis. **Arginine/Glycine could not be separated. ***ANOVA was applied to detect differences in total-AA, where significant difference was detected (*α* < 0.05), a Tukey HSD *post hoc* test was applied. Values with different superscript within a row are significantly different (*P* < 0.05).

The DIAA ratios were calculated and the DIAAS was calculated based on the DIAA ratios. The DIAA reference ratios are displayed in Table 3, and indicate whether all DIAA were present in the protein in adequate amounts to meet the requirements for adults set by FAO et al. (28). To calculate the ratios, the measured TAA levels were converted to mg/g protein for the different species as described in section 2.6.1. Scores above 1 indicate sufficient IAA levels, while ratios below 1 indicate insufficient levels. In addition, the DIAAS (%), was calculated, being the lowest amino acid ratio per species multiplied by 100 to convert the ratio to a percentage score. As indicated in Table 3, all amino acids show ratios above 1, despite methionine and cysteine (as combined values) in lemon sole (0.7), implying an excellent overall protein quality of the species. Cysteine and tryptophan were not analyzed in this study, which explains the low methionine + cysteine ratio, as it only consists of methionine. Ratios up to 2.5 for threonine were observed for thornback ray, megrim and lemon sole, pointing out the relevance of these species for a sufficient intake of indispensable amino acids. Moreover, DIAAS of over 100% were discovered for flounder (120%), megrim (120%), plaice (110%), and thornback ray (120%), stressing the high protein quality of the studied fish. Lemon sole shows a poorer DIAAS (70%), which is possibly due to the lack of data from cysteine.

**Table 3:** Average total digestible indispensable amino acid ratios and scores (DIAAS) for central fillets of flounder, lemon sole, megrim, plaice, and thornback ray based on recommendations for adults set by FAO et al. (28).

		Digestible indispensable amino acid ratios				
Amino acids	(28) Recommendations (mg/g protein)	Flounder	Lemon sole	Megrim	Plaice	Thornback ray
Histidine	15	1.3	1.5	1.5	1.4	1.3
Isoleucine	30	1.5	1.5	1.6	1.5	1.6
Leucine	59	1.5	1.4	1.5	1.5	1.5
Lysine	45	2.4	2.4	2.4	2.3	2.3
Methionine (+cys^*^)	22	1.6	0.7	1.3	1.4	1.6
Phenylalanine + tyrosine	38	2.0	2.4	2.0	1.1	2.0
Threonine	23	2.3	2.5	2.5	2.2	2.5
Tryptophan^*^	6	–	–	–	–	–
Valine	39	1.2	1.1	1.2	1.2	1.2
Total IAA	277	1.7	1.7	1.7	1.7	1.7
DIAAS (%)^**^		120%	70%	120%	110%	120%

*Cysteine/Tryptophan not measured in analysis. Scores above 1.0 indicate contents higher than recommendations (green cells), scores below 1.0 indicate content lower than recommendations (red cells). DIAAS (%) ** calculated by multiplying the lowest DIAA ratio by 100.

**Figure 2:**
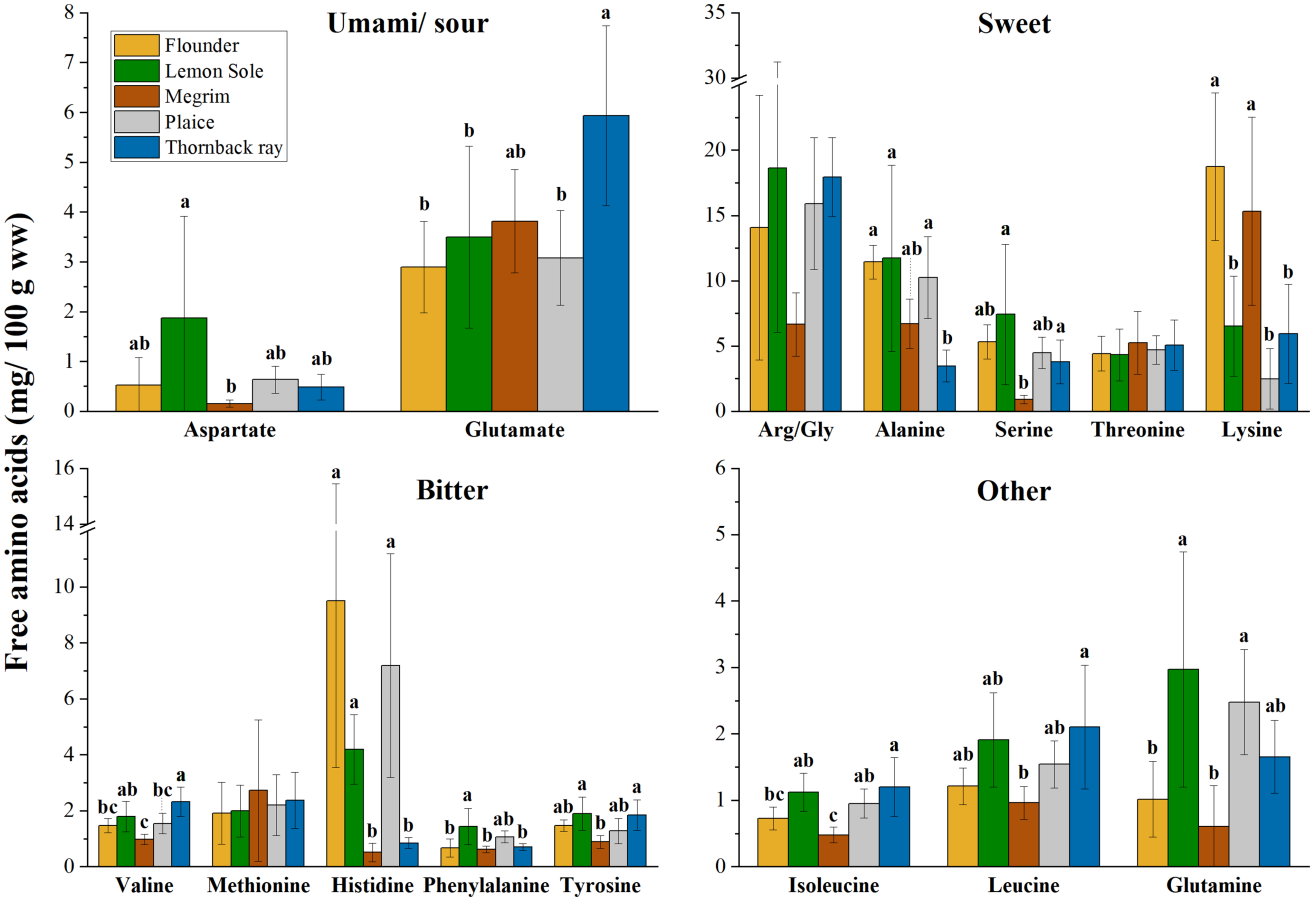
Free amino acids (FAA) in mg/100 g sample (ww), distribution grouped in taste perceptions umami/sour, sweet, bitter, and other. Error bars show SD. ANOVA was applied on species and each FAA; where significant difference between species was detected (*α* < 0.05), a Tukey HSD *post hoc* test was applied. Values with different letters (a, b) are significantly different (*p* < 0.05). Asparagine is not displayed due to shallow contents (>0.1 mg/100 g).

In addition to their nutritional importance, amino acids are associated with taste when occurring unbound in the form of free amino acids (FAA) in biological systems. Figure 2 shows the FAA distribution (mg/100 g sample ww) of the five investigated species. FAA are grouped according to their distinctive flavor as described by Fuke et al. (45), Kirimura et al. (46) and Sarower et al. (47). FAAs have been identified as essential taste contributors in seafood (45–47). Glutamic acid, in the form of glutamate, glycine, and alanine are commonly identified among the most important taste contributors. Glycine and alanine are linked to sweetness, while FAAs such as valine, arginine, and methionine are linked to bitter taste in seafood (47). Aspartate and glutamate both provide a sour taste. However, especially relevant in seafoods, these amino acids also give an umami taste in the presence of sodium salts, such as the familiar monosodium glutamate (MSG). Phenylalanine and tyrosine also have a bitter taste, but can enhance the umami flavor (47). Related to the present study, significant differences between species were found for all FAAs, but arginine/glycine (*p* = 0.114; Arg/Gly as combined values), threonine (*p* = 0.866) and methionine (*p* = 0.872). Lysine showed significant differences amongst the species (*p* < 0.001), with flounder and megrim having notably higher values. Flounder, lemon sole and plaice were identified to have significantly higher levels of histidine (*p* < 0.001), while megrim and thornback ray show comparatively low values of 0.51 and 0.84 mg/100 g sample, respectively. The most prevalent FAA are allocated in the group of “Sweet” amino acids with arginine/glycine having the highest content, followed by lysine and alanine.

Referring to the TAA and FAA distribution of the five investigated species, high variances within the species were observed, which is expressed by relatively large standard deviations as shown in Table 2 and Figure 2. External factors such as sex, maturity, and feeding behavior can influence the chemical composition of fish. Moreover, seasonality can play an important factor, as pointed out in a previous study on chemical composition of European plaice caught during three seasons by Kendler et al. (21).

### 3.3. Fatty acid composition

The following sections (section 3.3 and 3.4) highlight the potential of the studied species as sources for eicosapentaenoic acid (EPA) and docosahexaenoic acid (DHA) in human nutrition. Flounder, plaice and thornback ray were found to have significantly higher total PUFA compositions than megrim with 50.7, 47.2, and 49.3%, respectively, as can be seen in Table 4. Significant differences were also found in the amount of present DHA. Thornback ray shows considerable higher values (36.2%) comparing to the other species and profoundly lower values were observed in lemon sole (14.7%).

**Table 4:** Fatty acid composition (% of total fatty acids w w^−1^) of flounder, lemon sole, megrim, plaice, and thornback ray.

	Species					
Fatty acids	Flounder	Lemon sole	Megrim	Plaice	Thornback ray	
	*n* = 3^*^	*n* = 3^*^	*n* = 4^*^	*n* = 10	*n* = 5^*^	
**SFA**	%	%	%	%	%	*P*-value^**^
C14:0	1.58 ± 0.03^ab^	1.45 ± 0.3^ab^	2.68 ± 0.6^a^	2.71 ± 1.4^a^	0.29 ± 0.3	0.003
C15:0	0.52 ± 0.3	0.37 ± 0.3	0.34 ± 0.2	0.17 ± 0.3	0.06 ± 0.1	0.110
C16:0	20.9 ± 1.9^ab^	23.86 ± 6.2^a^	20.37 ± 3.0^ab^	17.1 ± 2.6^b^	26.0 ± 1.6^a^	<0.001
C17:0	0.66 ± 0.2^ab^	1.05 ± 0.1.0^a^	0.26 ± 0.2^ab^	0.17 ± 0.3^b^	0.29 ± 0.39^ab^	0.034
C18:0	5.52 ± 0.7^ab^	6.44 ± 1.1^a^	4.64 ± 1.4^ab^	3.50 ± 1.4^b^	5.06 ± 0.3^ab^	0.006
Ʃ SFA	29.19 ± 2.6^ab^	33.17 ± 6.4^a^	28.27 ± 3.3^ab^	23.64 ± 2.8^b^	31.66 ± 1.5^a^	<0.001
**MUFA**						
C14:1	0.04 ± 0.06^ab^	0.07 ± 0.1^ab^	0.22 ± 0.2^a^	0.03 ± 0.09^b^	0.00 ± 0.0^b^	0.041
C16:1 n7	2.88 ± 0.3^bc^	1.81 ± 1.6^bc^	4.78 ± 1.2^ab^	6.93 ± 1.9^a^	1.71 ± 0.09^c^	<0.001
C17:1	0.13 ± 0.2	0.39 ± 0.4	0.31 ± 0.2	0.18 ± 0.3	0.00 ± 0.0	0.273
C18:1 n7	2.37 ± 0.3	1.80 ± 1.6	2.66 ± 0.7	4.16 ± 2.4	3.55 ± 0.6	0.228
C18:1 n9	10.08 ± 2.3	12.19 ± 4.9	13.68 ± 2.9	8.45 ± 3.4	8.36 ± 0.7	0.055
C20:1	1.97 ± 0.3^ab^	0.77 ± 0.9^b^	5.26 ± 1.2^a^	4.89 ± 2.7^a^	1.27 ± 0.7^b^	0.003
C22:1	0.36 ± 0.6^ab^	0.12 ± 0.2^ab^	2.59 ± 2.0^ab^	2.91 ± 1.9^a^	0.00 ± 0.0^b^	0.008
Ʃ MUFA	17.82 ± 2.3^b^	17.16 ± 0.6^b^	29.50 ± 5.8^a^	27.53 ± 4.8^a^	14.89 ± 1.8^b^	<0.001
**PUFA**						
C16:2 n4	0.67 ± 0.2	0.73 ± 0.6	0.56 ± 0.4	0.40 ± 0.5	0.12 ± 0.3	0.351
C18:2 n6 (LA)	2.95 ± 1.8^a^	1.17 ± 1.0^ab^	0.88 ± 0.6^b^	0.40 ± 0.5^b^	1.49 ± 0.2^ab^	0.002
C18:3 n3	0.77 ± 0.3	0.43 ± 0.4	0.72 ± 0.5	0.21 ± 0.3	0.10 ± 0.2	0.023
C18:4 n3	0.39 ± 0.3	0.17 ± 0.3	0.51 ± 0.6	1.48 ± 1.9	0.00 ± 0.0	0.249
C20:2 n6	0.11 ± 0.2^ab^	0.39 ± 0.3^a^	0.20 ± 0.1^ab^	0.08 ± 0.1^ab^	0.00 ± 0.0^b^	0.037
C20:4 n6 (AA)	6.52 ± 1.6	8.94 ± 2.5	2.94 ± 1.8	4.11 ± 3.8	4.25 ± 0.6	0.07
C20:4 n3	0.16 ± 0.3	0.16 ± 0.3	0.62 ± 0.4	5.9 ± 7.0	0.14 ± 0.3	0.115
C20:5 n3 (EPA)	13.24 ± 2.6	14.93 ± 3.0	6.73 ± 2.3	10.07 ± 8.9	3.96 ± 0.7	0.119
C22:5 n3 (DPA)	2.09 ± 0.4	2.49 ± 2.2	2.31 ± 0.3	4.68 ± 5.6	3.11 ± 0.7	0.744
C22:6 n3 (DHA)	23.84 ± 4.4^bc^	14.72 ± 2.7^d^	24.80 ± 2.2^b^	19.83 ± 2.8^cd^	36.17 ± 1.4^a^	<0.001
Ʃ PUFA	50.74 ± 3.3^a^	44.12 ± 2.4^ab^	40.26 ± 4.0^b^	47.15 ± 4.0^a^	49.34 ± 2.0^a^	0.003
Ʃ n3	40.49 ± 5.2^abc^	32.9 ± 2.55^c^	35.67 ± 3.9^bc^	41.72 ± 4.4^ab^	43.48 ± 1.8^a^	0.005
Ʃ n6	9.58 ± 2.2	10.50 ± 1.4	4.02 ± 1.1	4.92 ± 4.2	5.74 ± 0.6	0.022
n3/n6	4.5 ± 1.8^bc^	3.2 ± 0.6^c^	9.3 ± 2.4^a^	6.1 ± 2.3^abc^	7.6 ± 0.9^ab^	0.003
Ʃ Others	2.24 ± 3.9	5.55 ± 5.3	1.97 ± 2.1	1.67 ± 2.9	4.12 ± 1.4	0.303

Results presented as mean values ± SD. *Samples were merged in order to provide enough lipid phase to analyze for fatty acid composition. **ANOVA was applied to detect differences in fatty acid composition; where significant difference was detected (*α* < 0.05), a Tukey HSD *post hoc* test was applied. Values with different superscript (a, b) within a row are significantly different (*P* < 0.05).

Although the studied fish are categorized as lean species, the fatty acid composition is of high importance. Health promoting effects due to prevention of cardiovascular diseases (CVD), tumor cell proliferation and inflammation processes as well as beneficial effects on brain, retina and neurodevelopment in children are primarily attributed to eicosapentaenoic acid (EPA) and docosahexaenoic acid (DHA) (9).

### 3.4. Nutritional value and essential elements

**Figure 3:**
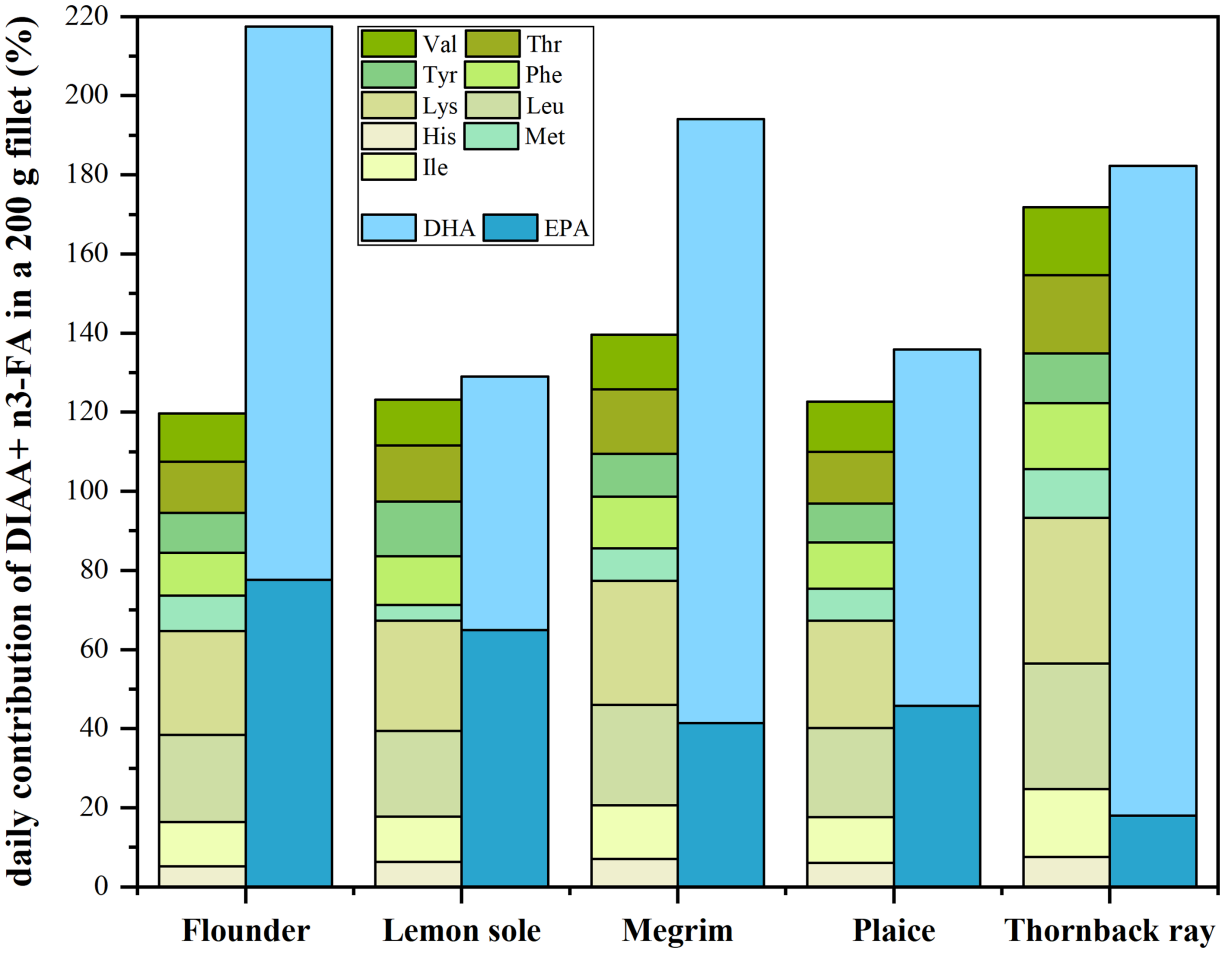
Given in green colors: average contribution of all DIAA to daily requirements for an 80 kg adult in a 200 g fillet portion by FAO, WHO (11); given in blue colors: average contribution of a 200 g fillet portion to cover the daily recommended intake of 250 mg DHA + EPA by EFSA (48).

The total EPA and DHA intake when consuming the five investigated species was calculated to highlight how the species contribute to providing these indispensable FAs to human diet. The contribution to the daily recommendations of 250 mg EPA and DHA by the EFSA Panel on Dietetic Products et al. (48) are shown in Figure 3. Moreover, the average contribution of all IAA given as DIAAS by FAO (24) including the daily requirements of IAA for an 80 g adult as proposed by FAO et al. (28) are shown in Figure 3. The values refer to a portion size of 200 g as recommended in a report on dinner serving sizes of foods by the Norwegian Food Authority (Mattilsynet) and were used for the calculations (49).

Figure 3 emphasizes on the nutritional value of the studied fish related to the content of DIAA as well as EPA and DHA in a 200 g fillet portion. The green bars show that the total required daily amount of DIAA for an 80 kg adult are met by all species. A fillet of 200 g of all species covers more than 120% of the total required DIAA, with thornback ray fulfilling the requirements to 172%. Nevertheless, to fully cover the daily intake of every individual indispensable amino acid (100%), a larger portion size than 200 g is needed for all investigated fish species. The daily recommended intake of, e.g., valine was restrictively covered in megrim (69%), hence a portion size of 371 g would be necessary to cover the daily demand of this particular amino acid (100%). Furthermore, valine is also the restrictive amino acid for thornback ray and flounder, where 54 and 61% of the daily demand is covered by a 200 g portion, respectively. In plaice, phenylalanine (53%) and in lemon sole, methionine (33%) are not fully covered with regards to a portion size of 200 g.

With regards to EPA and DHA, all investigated fish species contribute significantly to the daily suggested intake of 250 mg by EFSA (48). The relative contribution of DHA and EPA in 200 g fillets is shown in dark/light blue shading and was converted to mg/100 g edible portion using the conversion factors proposed by Weihrauch et al. (29) as described in section 2.6.2. Even tough being lean species, a 200 g portion of all of the five species contributes in average to more than 100% of the recommended average daily intake (AI) of EPA and DHA set by EFSA (48). The highest contribution was found in a 200 g portion of flounder (217%), followed by megrim (194%), thornback ray (182%), plaice (136%) and lemon sole (129%). With regards to the weekly recommended intake of 1.75 g EPA + DHA (250 mg × 7) this would mean a consumption of 3.2 portions of 200 g flounder, 3.6 of megrim, 3.8 of thornback ray, 5.2 of plaice and 5.4 portions of lemon sole, respectively. These results are highly relevant, as lean fish is usually not associated with providing sufficient levels of n3 fatty acids and the focus for covering n3 fatty acids was previously put on fatty fish such as salmon or trout in the past (50). All investigated species contain higher relative amounts of DHA, despite lemon sole, which has a 50:50 share of EPA and DHA.

Marine fish are good sources for both macro and trace elements, including minerals like calcium, magnesium or selenium, being vital for human health. All fish contain sufficient amounts of potassium (K) and magnesium as shown in Table 5. Significant differences between species were observed for the elements manganese (*p* < 0.001), magnesium (*p* = 0.002) and iron (*p* = 0.047). High values in selenium, ranging from 0.25 mg kg^−1^ in thornback ray to 0.49 mg kg^−1^ in lemon sole were found in this study. Compared to the study of Karl et al. (34) on different flatfish, selenium values ranging from 0.13 to 0.31 mg kg^−1^ were reported. When setting dietary recommendations, the dietary reference value (DRV) is used. The DRV in this study refers to either the average requirement (AR), the population reference intake (PRI) or the adequate intake (AI), depending on the available data from the expert panel on Dietetic Products, Nutrition and Allergies from EFSA (51–60). Even though no significant difference (*p* = 0.213) in selenium content between species was detected, an effect on the contribution to meet the DRV is visible, given as % of DRV. Hence, a 200 g fillet of lemon sole covers the selenium intake to 140%, whereas a 200 g fillet of thornback ray reaches only 74.3% of the daily selenium coverage. Moreover, all five species are a good source of potassium, covering around 20% of the DRV. Differences in species are visible for the magnesium contribution, where a 200 g portion of plaice covers 10% of the DRV and megrim contributes up to 16% of the daily recommended intake.

**Table 5:** Essential elements and nutritional contribution of a 200 g portion of fillet of flounder, lemon sole, megrim, plaice, and thornback ray.

		**Species**
		Flounder (*n* = 2)			Lemon sole (*n* = 2)			Megrim (*n* = 2)			Plaice (*n* = 6)			Thornback ray (*n* = 2)			
Element	DRV (mg/day)	μg kg^−1^	EDI	% DRV	μg kg^−1^	EDI	% DRV	μg kg^−1^	EDI	% DRV	μg kg^−1^	EDI	% DRV	μg kg^−1^	EDI	% DRV	P-value*
																	
Mn	3.0 (51)	70.5 ± 5.5^bc^	0.014	0.47	94.8 ± 2.2^ab^	0.019	0.63	107 ± 16.6^ab^	0.021	0.71	39.8 ± 14.5^□c^	0.008	0.27	145.8 ± 27.1^a^	0.029	0.97	<0.001
Mo	0.065 (52)	0.7 ± 0.06	1×10^−4^	0.22	1.2 ± 0.38	3×10^−4^	0.38	0.06 ± 0.08	1×10^−5^	0.012	0.87 ± 0.4^□^	2×10^−4^	0.28	1.82 ± 1.5	4×10^−4^	0.6	0.114
Co	/	2.5 ± 0.15	5×10^−4^		2.4 ± 1.12	5×10^−4^		0.52 ± 0.12	1×10^−4^		2.52 ± 1.1^□^	5×10^−4^		1.08 ± 0.23	2×10^−4^		0.098
		mg kg^−1^			mg kg^−1^			mg kg^−1^			mg kg^−1^			mg kg^−1^			
K	3,500 (53)	3,622 ± 315	724.4	20.7	3,411 ± 409	682.2	19.5	3,689 ± 4.3	737.8	21.1	3,493 ± 507	698.6	20.0	3,495 ± 154	699	20.0	0.847
Na	2000 (54)	919 ± 71.4	183.8	9.2	730 ± 99.9	145.9	7.3	644 ± 201	128.9	6.4	1,085 ± 248	217	10.9	774.5 ± 86.0	154.9	7.7	0.191
Mg	300–350 (55)	244 ± 28.2^ab^	48.7	13.9	218 ± 34.6^bc^	43.5	12.4	280 ± 6.7^a^	56.0	16.0	188.7 ± 7.8^c^	37.74	10.8	232.1 ± 2^abc^	46.4	13.3	0.002
Ca	950–1,000 (56)	151 ± 30.2	30.24	3.0	149 ± 35.7	29.8	3.0	146 ± 28.6	29.12	2.9	153.7 ± 59.1	30.74	3.1	172.5 ± 107	34.5	3.5	0.993
Fe	11–16 (57)	0.8 ± 0.24	0.16	1.0	0.83 ± 0.25	0.17	1.0	0.74 ± 0.23	0.15	0.9	0.94 ± 0.25^□^	0.188	1.2	1.65 ± 0.5	0.33	2.1	0.047
Zn	12.7–16.3 (58)	3.7 ± 0.2	0.74	4.5	3.18 ± 0.16	0.64	3.9	3.75 ± 0.08	0.75	4.6	3.90 ± 0.48^□^	0.78	4.8	3.23 ± 0.3	0.65	4.0	0.172
Se	0.070 (59)	0.4 ± 0.02	0.07	100	0.49 ± 0.01	0.098	140	0.40 ± 0.02	0.08	114.3	0.40 ± 0.12^□^	0.08	114	0.26 ± 0.0	0.052	74.3	0.213
Cu	1.3–1.6 (60)	0.2 ± 0.06^ab^	0.034	2.1	0.16 ± 0.01^ab^	0.032	2	0.2 ± 0.002^a^	0.038	2.4	0.11 ± 0.02^□b^	0.022	1.4	0.2 ± 0.03^ab^	0.032	2	0.031

*n* = pooled samples constituting of multiple individuals per sample. Results presented as mean values ± SD; DRV, dietary reference values; EDI, estimated daily intake, mg/200 g fillet. ^□^Results extracted from a previous study by Kendler et al. (21). *ANOVA was applied to detect differences in trace elements, where significant difference was detected (*α* < 0.05), a Tukey HSD post hoc test was applied. Values with different superscript (^a,b^) within a row are significantly different (*p* < 0.05). DRV is expressed as PRI (population reference intake) or AI (adequate intake), dependent on the relative scientific opinion from EFSA as given in the references (51–60). For EDI calculation, average DRV values of Mg, Fe, Zn, and Cu were used.

### 3.5. Contaminants

Being demersal fish species, *Pleuronoectiformes* and *Rajiformes* are more likely to accumulate PCBs and hazardous trace elements than other fish species (13). Therefore, when frequently eating flatfish or ray, the matter of food safety must be considered. However, both intrinsic and environmental variables have a role in the bioaccumulation of hazardous as well as beneficial compounds (31). Individuals different in sizes and sexes, exhibit different concentrations in trace elements and contaminants, due to a variety in habitat and migration behaviors. In this study several toxic trace elements were determined (Table 6). The analysis on PCBs, including both non-dioxin and dioxin-like PCB congeners, show values lower than the detection limit (LOD) for flounder, lemon sole, megrim, and thornback ray. Hence no significant accumulation of any of the investigated PCB congeners were detected in those four species. Concerning plaice, traces of PCB 3, 52, 101, 118, 138, 153, and 180 were detected, as previously reported in the study of Kendler et al. (21) that looked into seasonal differences. A reason for the lower PCB values compared to plaice could be the lower fat content as well as overall lower fish size in the three other flatfish and the thornback ray. With regards to toxic trace elements, significantly different values between the species were detected for the elements chromium (*p* = 0.002), nickel (*p* < 0.001), cadmium (*p* < 0.001) and lead (*p* < 0.001). The highest accumulation of chromium and cadmium were found in thornback ray with 56.36 ± 29.12 μg kg^−1^ and 0.58 ± 0.05 μg kg^−1^ respectively. Large varieties within species can be seen for all elements, with particular great SD in the mercury content of plaice (112.9 ± 54.1 μg kg^−1^) and thornback ray (174.37 ± 169 μg kg^−1^). Despite differences between species as well as individuals, the maximum levels of cadmium (0.1 mg kg^−1^), lead (0.3 mg kg^−1^) and mercury (0.5 mg kg^−1^) set by the EC (61) are not exceeded. When considering arsenic as potential hazardous component, organic and inorganic arsenic must be differentiated. Inorganic arsenic is the toxic form and according to Sloth et al. (62) a maximum of 1% of total arsenic in marine species is found in the form of hazardous arsenite and arsenate. The calculations in the previous study of Kendler et al. (21) on arsenic content in European plaice were followed for the other four species in this study. Considering the suggestion of 1% inorganic arsenic (58), it is safe to consume the recommended portion size by the Norwegian Food Authority (49) of 200 g of each of the five investigated species.

**Table 6:** Toxic trace elements of flounder, lemon sole, megrim, plaice, and thornback ray.

	Season					
Toxic trace elements	Flounder	Lemon sole	Megrim	Plaice^□^	Thornback ray	
	*n* = 2	*n* = 2	*n* = 2	*n* = 6	*n* = 2	*P*-value*
	μg kg^−1^	μg kg^−1^	μg kg^−1^	μg kg^−1^	μg kg^−1^	
V	5.54 ± 4.41	10.20 ± 10.57	2.54 ± 1.69	12.4 ± 16.3	0.67 ± 0.05	0.756
Cr	14.71 ± 2.99^bc^	37.89 ± 6.28^ab^	12.56 ± 2.63^bc^	7.6 ± 2.1^c^	56.36 ± 29.12^a^	0.002
Ni	16.52 ± 2.4^a^	9.29 ± 0.06^b^	8.92 ± 0.8^b^	2.01 ± 0.6^c^	10.75 ± 1.07^b^	<0.001
Ag	0.24 ± 0.1	0.35 ± 0.49	0.13 ± 0.03	0.19 ± 0.1	0.51 ± 0.02	0.316
Cd	0.19 ± 0.01^b^	0.13 ± 0.03^b^	0.12 ± 0.05^b^	0.17 ± 0.09^b^	0.58 ± 0.05^a^	<0.001
Pb	2.42 ± 0.67^ab^	2.98 ± 0.17^a^	3.50 ± 0.37^a^	0.81 ± 0.5^c^	1.20 ± 0.2^bc^	<0.001
Hg	39.78 ± 1.74	58.05 ± 2.29	70.77 ± 10.69	112.9 ± 54.1	174.37 ± 169	0.350
	mg kg^−1^	mg kg^−1^	mg kg^−1^	mg kg^−1^	mg kg^−1^	
As	19.87 ± 7.05	105.3 ± 65.6	3.51 ± 0.63	58.9 ± 28.3	29.94 ± 9.33	0.051
Ʃ toxic elements	19.95 ± 7.1	105.4 ± 65.6	3.60 ± 0.6	59.05 ± 28.4	30.18 ± 9.5	

*n* = pooled samples constituting of multiple individuals per sample. Results presented as mean values ± SD. ^□^Results extracted from a previous study by Kendler et al. (21). *ANOVA was applied to detect differences in trace elements and PCBs; where significant difference was detected (*α* < 0.05), a Tukey HSD *post hoc* test was applied. Values with different superscript (^a, b^) within a row are significantly different (*P* < 0.05).

## 4. Conclusion

This study highlighted the nutritional composition of flounder, lemon sole, megrim, plaice, and thornback ray. The distribution and contribution of DIAA and the two main n3 fatty acids EPA and DHA show remarkable nutritional quality in all five species. A 200 g fillet portion of each of the five species covers the total DIAA and the recommended average daily intake of n3 fatty acids for an adult person. The nutritional score, emphasizing on DIAA and n3-fatty acids, can be regarded as profitable with good overall quality of all five fish. This study emphasized on the benefits of consuming these five species, mainly in the form of n3-fatty acids, DIAA, and essential minerals, but also investigated potential hazardous components. Potential risk factors in the form of PCBs and toxic trace elements were analyzed and have shown only minor bioaccumulation of single elements below the suggested upper intake limits. In conclusion, our study provides important insights into the nutritional profile of five underutilized fish species in Norway. However, it is important to note that there were some limitations in our study, including an incomplete TAA profile analysis, by not covering tryptophan and cysteine, as well as a potential overestimation of the protein content due to the chosen conversion factor. To further improve the understanding of the amino acid composition and total protein content, future studies should include cysteine analysis and focus on evaluating species-specific conversion factors Future work should also put a stronger focus on assessing the risks and benefits of these fish that come with increased consumption. This is necessary to promote a safe consumption and integrate these fish, which have not yet been considered commercially in Norway, into the diet.

## Data availability statement

The raw data supporting the conclusions of this article will be made available by the authors, without undue reservation.

## Author contributions

SK: conceptualization, methodology, formal analysis, investigation, and writing – original draft. FT: investigation and writing – original draft. AJ: conceptualization, methodology, and supervision. JL: conceptualization, methodology, supervision, project administration, and funding acquisition. All authors contributed to the article and approved the submitted version.

## Funding

This work was funded by the OPTiMAT project from the Norwegian University of Science and Technology (NTNU), Trondheim.

## Conflict of interest

The authors declare that the research was conducted in the absence of any commercial or financial relationships that could be construed as a potential conflict of interest.

## Publisher’s note

All claims expressed in this article are solely those of the authors and do not necessarily represent those of their affiliated organizations, or those of the publisher, the editors and the reviewers. Any product that may be evaluated in this article, or claim that may be made by its manufacturer, is not guaranteed or endorsed by the publisher.

## Footnotes

^1^www.minitab.com
